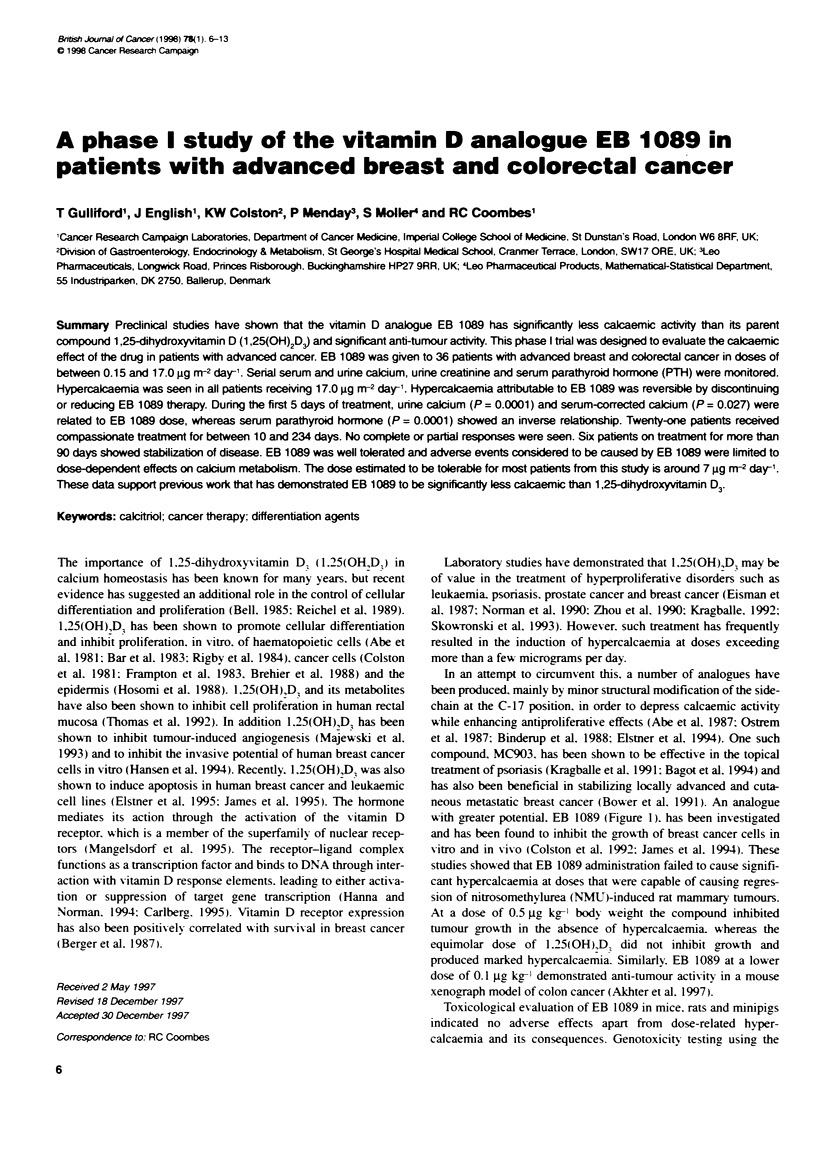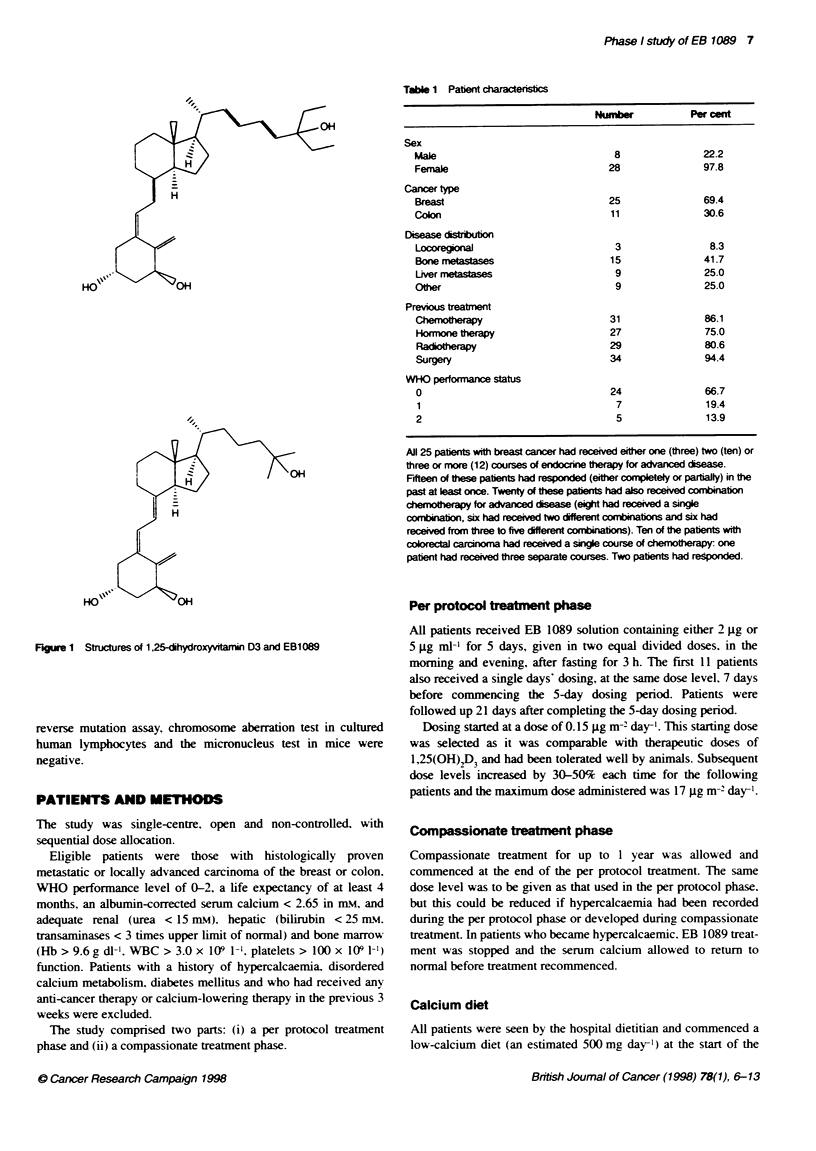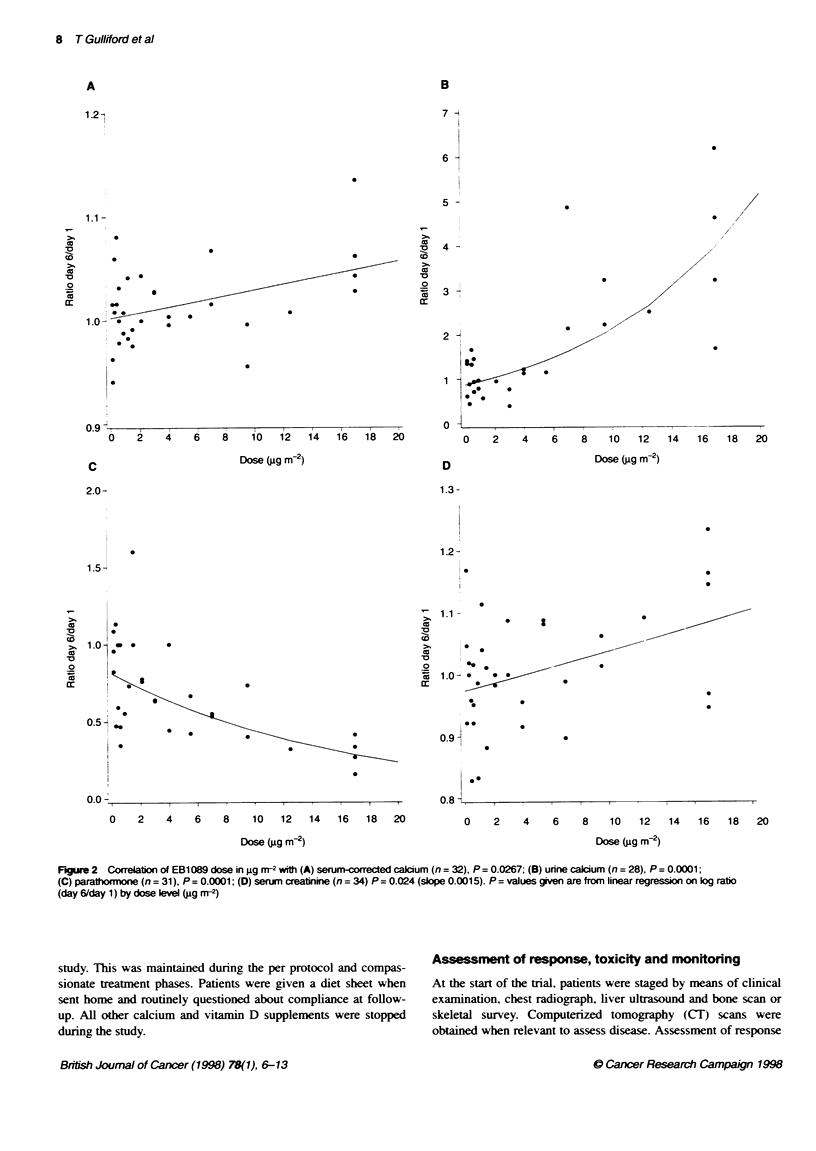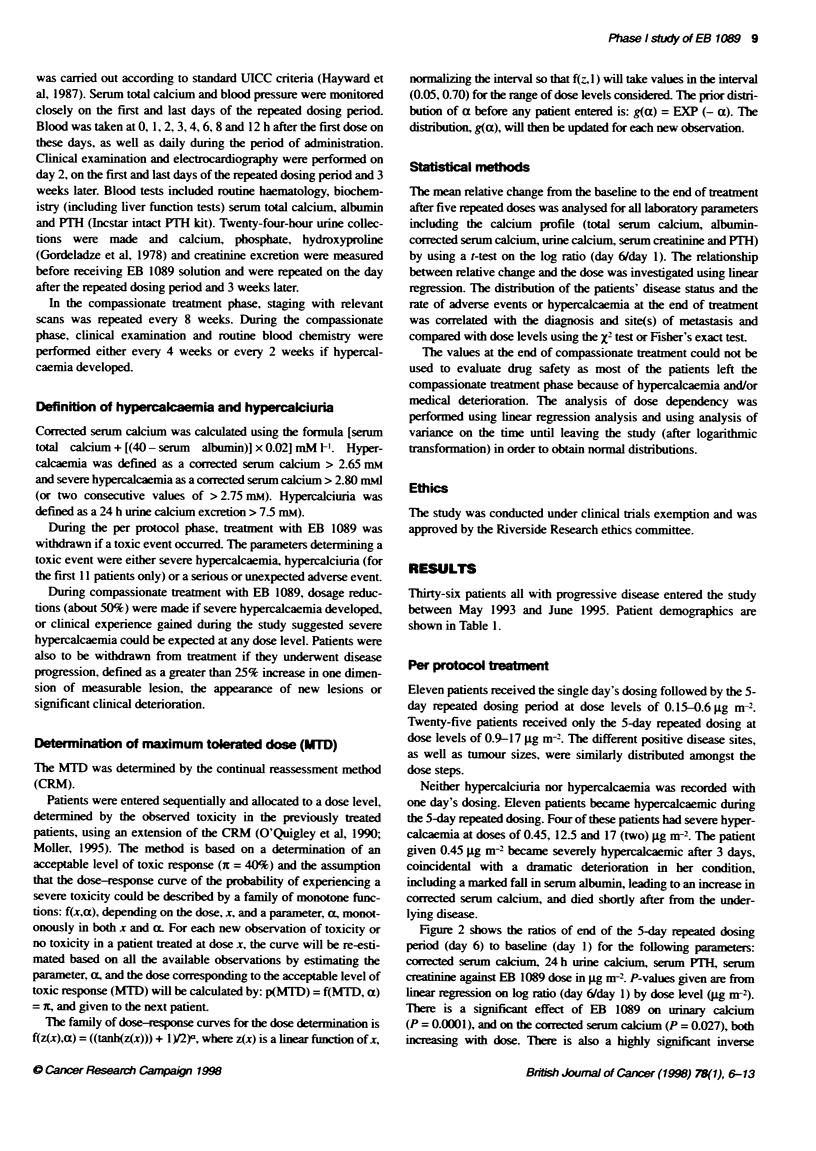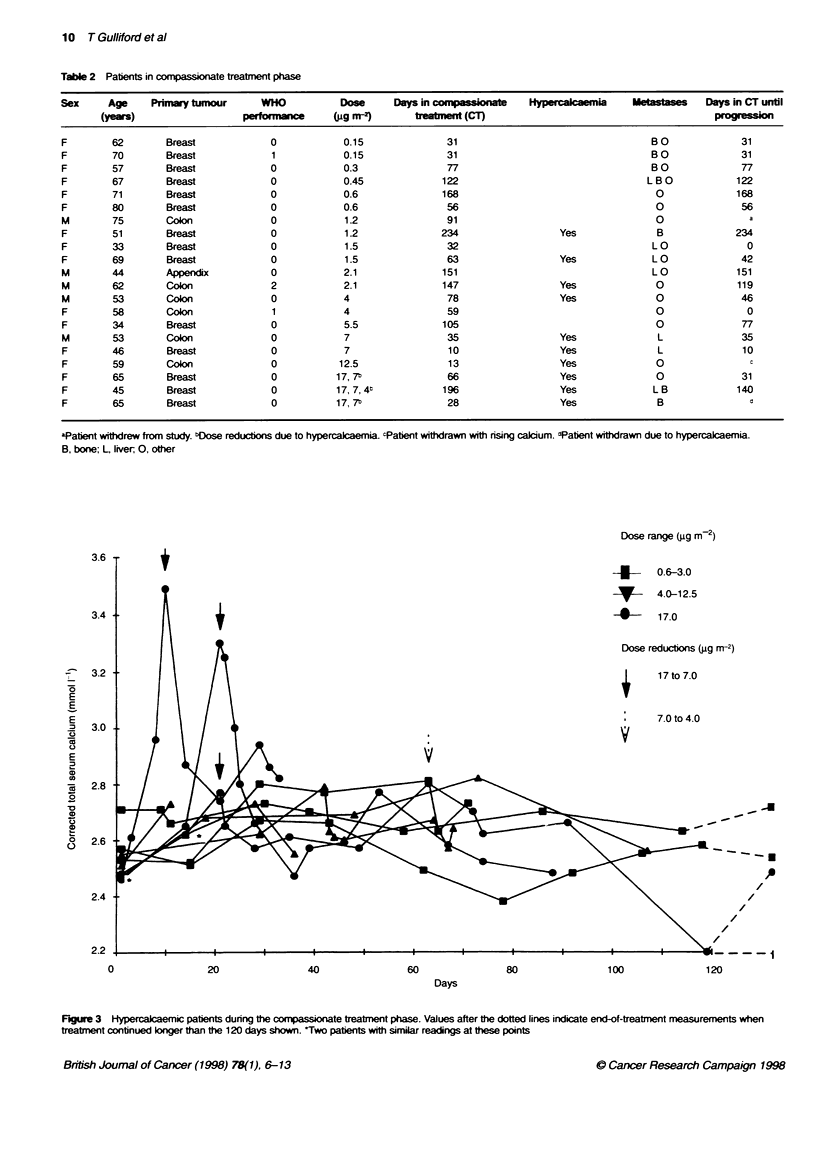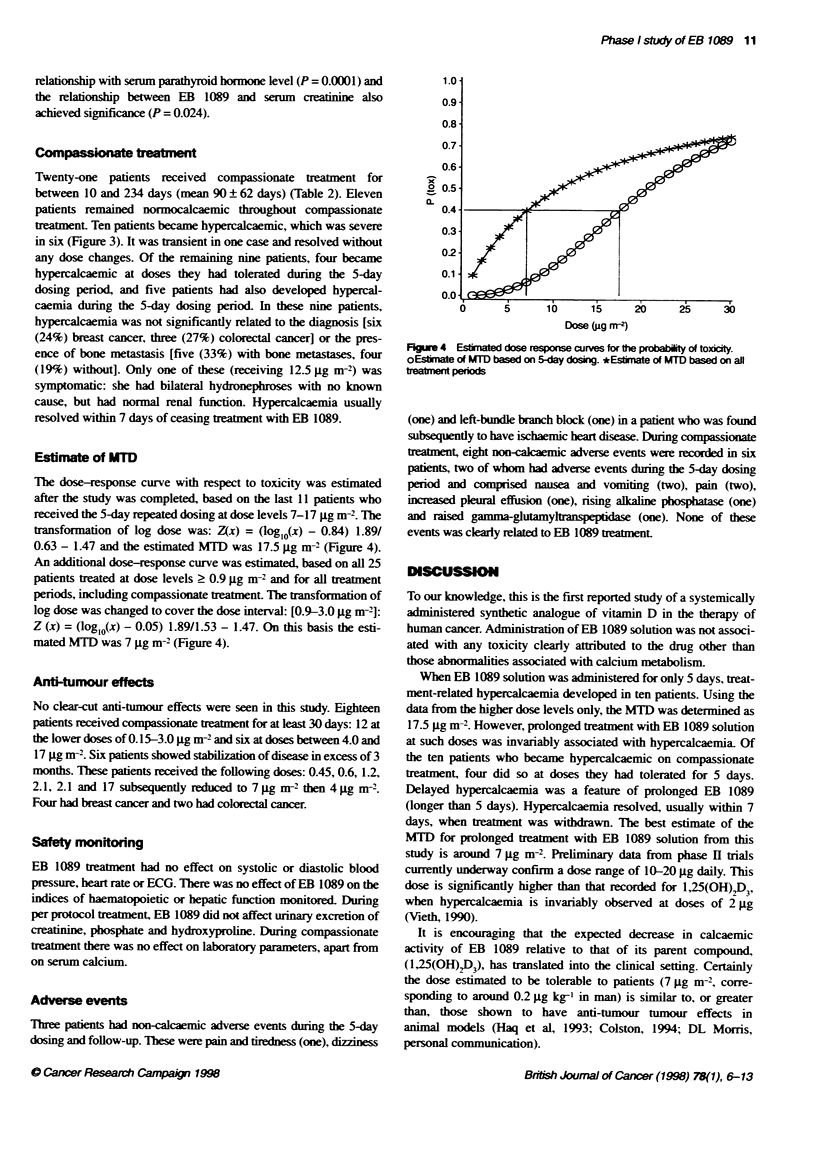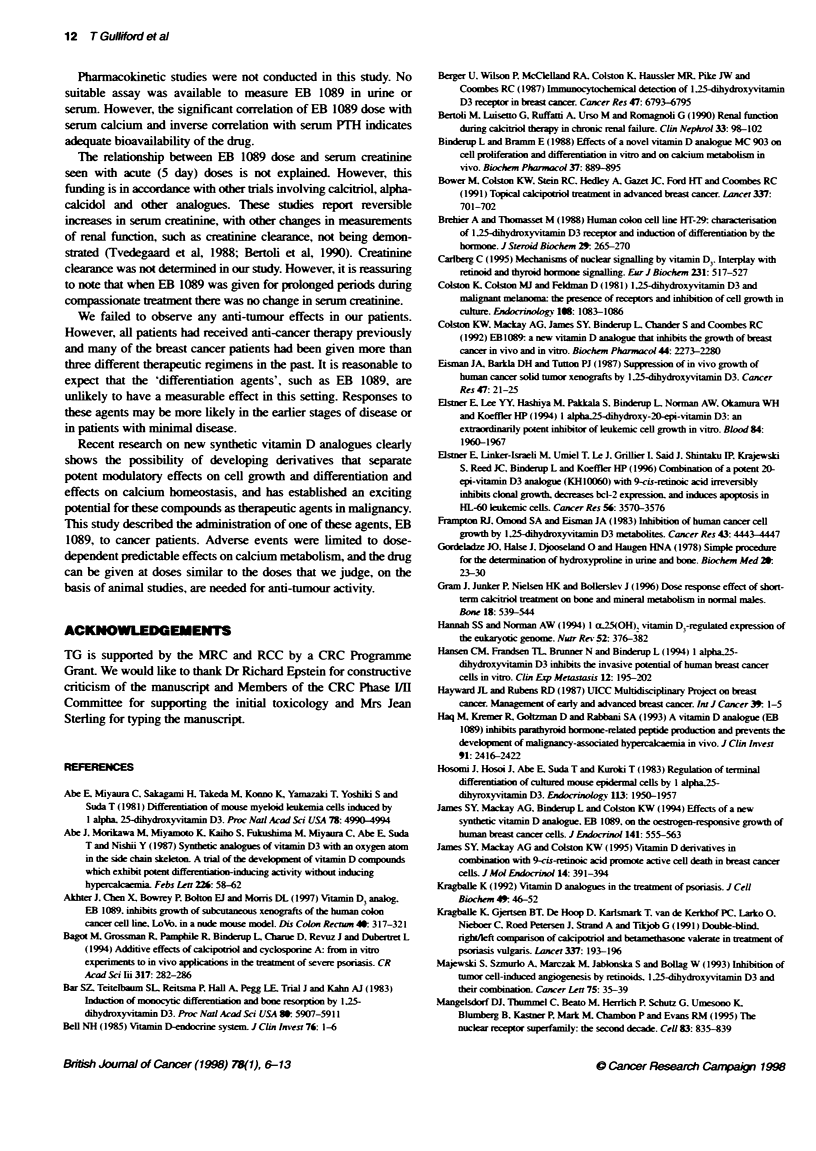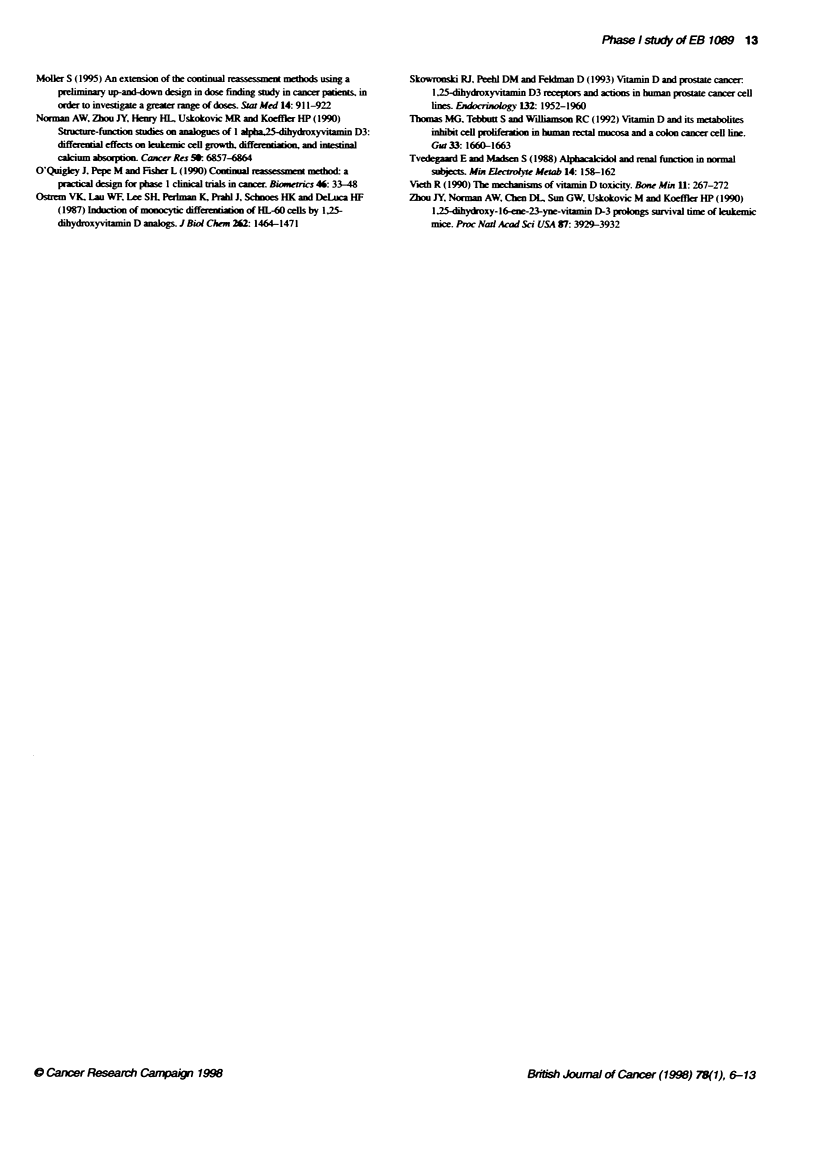# A phase I study of the vitamin D analogue EB 1089 in patients with advanced breast and colorectal cancer.

**DOI:** 10.1038/bjc.1998.434

**Published:** 1998-07

**Authors:** T. Gulliford, J. English, K. W. Colston, P. Menday, S. Moller, R. C. Coombes

**Affiliations:** Cancer Research Campaign Laboratories, Department of Cancer Medicine, Imperial College School of Medicine, London, UK.

## Abstract

Preclinical studies have shown that the vitamin D analogue EB 1089 has significantly less calcaemic activity than its parent compound 1,25-dihydroxyvitamin D (1,25(OH)2D3) and significant anti-tumour activity. This phase I trial was designed to evaluate the calcaemic effect of the drug in patients with advanced cancer. EB 1089 was given to 36 patients with advanced breast and colorectal cancer in doses of between 0.15 and 17.0 microg m(-2) day(-1). Serial serum and urine calcium, urine creatinine and serum parathyroid hormone (PTH) were monitored. Hypercalcaemia was seen in all patients receiving 17.0 microg m(-2) day(-1). Hypercalcaemia attributable to EB 1089 was reversible by discontinuing or reducing EB 1089 therapy. During the first 5 days of treatment, urine calcium (P = 0.0001) and serum-corrected calcium (P = 0.027) were related to EB 1089 dose, whereas serum parathyroid hormone (P = 0.0001) showed an inverse relationship. Twenty-one patients received compassionate treatment for between 10 and 234 days. No complete or partial responses were seen. Six patients on treatment for more than 90 days showed stabilization of disease. EB 1089 was well tolerated and adverse events considered to be caused by EB 1089 were limited to dose-dependent effects on calcium metabolism. The dose estimated to be tolerable for most patients from this study is around 7 microg m(-2) day(1). These data support previous work that has demonstrated EB 1089 to be significantly less calcaemic than 1,25-dihydroxyvitamin D3.


British Journal of Cancer (1998) 78(1), 6–13
© 1998 Cancer Research Campaign

### A phase I study of the vitamin D analogue EB 1089 in patients with advanced breast and colorectal cancer

**T Gulliford[1], J English[1], KW Colston[2], P Menday[3], S Moller[4] and RC Coombes[1]**

[1]Cancer Research Campaign Laboratories, Department of Cancer Medicine, Imperial College School of Medicine, St Dunstan's Road, London W6 8RF, UK; [2]Division of Gastroenterology, Endocrinology & Metabolism, St George's Hospital Medical School, Cranmer Terrace, London, SW17 ORE, UK; [3]Leo Pharmaceuticals, Longwick Road, Princes Risborough, Buckinghamshire HP27 9RR, UK; [4]Leo Pharmaceutical Products, Mathematical-Statistical Department, 55 Industriparken, DK 2750, Ballerup, Denmark

**Summary** Preclinical studies have shown that the vitamin D analogue EB 1089 has significantly less calcaemic activity than its parent compound 1,25-dihydroxyvitamin D (1,25$(OH)_2D_3$) and significant anti-tumour activity. This phase I trial was designed to evaluate the calcaemic effect of the drug in patients with advanced cancer. EB 1089 was given to 36 patients with advanced breast and colorectal cancer in doses of between 0.15 and 17.0 μg $m^{-2}$ $day^{-1}$. Serial serum and urine calcium, urine creatinine and serum parathyroid hormone (PTH) were monitored. Hypercalcaemia was seen in all patients receiving 17.0 μg $m^{-2}$ $day^{-1}$. Hypercalcaemia attributable to EB 1089 was reversible by discontinuing or reducing EB 1089 therapy. During the first 5 days of treatment, urine calcium ($P$ = 0.0001) and serum-corrected calcium ($P$ = 0.027) were related to EB 1089 dose, whereas serum parathyroid hormone ($P$ = 0.0001) showed an inverse relationship. Twenty-one patients received compassionate treatment for between 10 and 234 days. No complete or partial responses were seen. Six patients on treatment for more than 90 days showed stabilization of disease. EB 1089 was well tolerated and adverse events considered to be caused by EB 1089 were limited to dose-dependent effects on calcium metabolism. The dose estimated to be tolerable for most patients from this study is around 7 μg $m^{-2}$ $day^{-1}$. These data support previous work that has demonstrated EB 1089 to be significantly less calcaemic than 1,25-dihydroxyvitamin $D_3$.

**Keywords:** calcitriol; cancer therapy; differentiation agents

The importance of 1,25-dihydroxyvitamin $D_3$ (1,25$(OH_2D_3)$ in calcium homeostasis has been known for many years, but recent evidence has suggested an additional role in the control of cellular differentiation and proliferation (Bell, 1985; Reichel et al, 1989). 1,25$(OH)_2D_3$ has been shown to promote cellular differentiation and inhibit proliferation, in vitro, of haematopoietic cells (Abe et al, 1981; Bar et al, 1983; Rigby et al, 1984), cancer cells (Colston et al, 1981; Frampton et al, 1983, Brehier et al, 1988) and the epidermis (Hosomi et al, 1988). 1,25$(OH)_2D_3$ and its metabolites have also been shown to inhibit cell proliferation in human rectal mucosa (Thomas et al, 1992). In addition 1,25$(OH)_2D_3$ has been shown to inhibit tumour-induced angiogenesis (Majewski et al, 1993) and to inhibit the invasive potential of human breast cancer cells in vitro (Hansen et al, 1994). Recently, 1,25$(OH)_2D_3$ was also shown to induce apoptosis in human breast cancer and leukaemic cell lines (Elstner et al, 1995; James et al, 1995). The hormone mediates its action through the activation of the vitamin D receptor, which is a member of the superfamily of nuclear receptors (Mangelsdorf et al, 1995). The receptor–ligand complex functions as a transcription factor and binds to DNA through interaction with vitamin D response elements, leading to either activation or suppression of target gene transcription (Hanna and Norman, 1994; Carlberg, 1995). Vitamin D receptor expression has also been positively correlated with survival in breast cancer (Berger et al, 1987).

*Received 2 May 1997*
*Revised 18 December 1997*
*Accepted 30 December 1997*

*Correspondence to:* RC Coombes

Laboratory studies have demonstrated that 1,25$(OH)_2D_3$ may be of value in the treatment of hyperproliferative disorders such as leukaemia, psoriasis, prostate cancer and breast cancer (Eisman et al, 1987; Norman et al, 1990; Zhou et al, 1990; Kragballe, 1992; Skowronski et al, 1993). However, such treatment has frequently resulted in the induction of hypercalcaemia at doses exceeding more than a few micrograms per day.

In an attempt to circumvent this, a number of analogues have been produced, mainly by minor structural modification of the side-chain at the C-17 position, in order to depress calcaemic activity while enhancing antiproliferative effects (Abe et al, 1987; Ostrem et al, 1987; Binderup et al, 1988; Elstner et al, 1994). One such compound, MC903, has been shown to be effective in the topical treatment of psoriasis (Kragballe et al, 1991; Bagot et al, 1994) and has also been beneficial in stabilizing locally advanced and cutaneous metastatic breast cancer (Bower et al, 1991). An analogue with greater potential, EB 1089 (Figure 1), has been investigated and has been found to inhibit the growth of breast cancer cells in vitro and in vivo (Colston et al, 1992; James et al, 1994). These studies showed that EB 1089 administration failed to cause significant hypercalcaemia at doses that were capable of causing regression of nitrosomethylurea (NMU)-induced rat mammary tumours. At a dose of 0.5 μg $kg^{-1}$ body weight the compound inhibited tumour growth in the absence of hypercalcaemia, whereas the equimolar dose of 1,25$(OH)_2D_3$ did not inhibit growth and produced marked hypercalcaemia. Similarly, EB 1089 at a lower dose of 0.1 μg $kg^{-1}$ demonstrated anti-tumour activity in a mouse xenograph model of colon cancer (Akhter et al, 1997).

Toxicological evaluation of EB 1089 in mice, rats and minipigs indicated no adverse effects apart from dose-related hypercalcaemia and its consequences. Genotoxicity testing using the

**Figure 1** Structures of 1,25-dihydroxyvitamin D3 and EB1089

reverse mutation assay, chromosome aberration test in cultured human lymphocytes and the micronucleus test in mice were negative.

### PATIENTS AND METHODS

The study was single-centre, open and non-controlled, with sequential dose allocation.

Eligible patients were those with histologically proven metastatic or locally advanced carcinoma of the breast or colon, WHO performance level of 0–2, a life expectancy of at least 4 months, an albumin-corrected serum calcium < 2.65 in mM, and adequate renal (urea < 15 mM), hepatic (bilirubin < 25 mM, transaminases < 3 times upper limit of normal) and bone marrow (Hb > 9.6 g $dl^{-1}$, WBC > 3.0 × $10^9$ $l^{-1}$, platelets > 100 × $10^9$ $l^{-1}$) function. Patients with a history of hypercalcaemia, disordered calcium metabolism, diabetes mellitus and who had received any anti-cancer therapy or calcium-lowering therapy in the previous 3 weeks were excluded.

The study comprised two parts: (i) a per protocol treatment phase and (ii) a compassionate treatment phase.

**Table 1** Patient characteristics

| | Number | Per cent |
|---|---|---|
| Sex | | |
| Male | 8 | 22.2 |
| Female | 28 | 97.8 |
| Cancer type | | |
| Breast | 25 | 69.4 |
| Colon | 11 | 30.6 |
| Disease distribution | | |
| Locoregional | 3 | 8.3 |
| Bone metastases | 15 | 41.7 |
| Liver metastases | 9 | 25.0 |
| Other | 9 | 25.0 |
| Previous treatment | | |
| Chemotherapy | 31 | 86.1 |
| Hormone therapy | 27 | 75.0 |
| Radiotherapy | 29 | 80.6 |
| Surgery | 34 | 94.4 |
| WHO performance status | | |
| 0 | 24 | 66.7 |
| 1 | 7 | 19.4 |
| 2 | 5 | 13.9 |

All 25 patients with breast cancer had received either one (three) two (ten) or three or more (12) courses of endocrine therapy for advanced disease. Fifteen of these patients had responded (either completely or partially) in the past at least once. Twenty of these patients had also received combination chemotherapy for advanced disease (eight had received a single combination, six had received two different combinations and six had received from three to five different combinations). Ten of the patients with colorectal carcinoma had received a single course of chemotherapy: one patient had received three separate courses. Two patients had responded.

### Per protocol treatment phase

All patients received EB 1089 solution containing either 2 μg or 5 μg $ml^{-1}$ for 5 days, given in two equal divided doses, in the morning and evening, after fasting for 3 h. The first 11 patients also received a single days' dosing, at the same dose level, 7 days before commencing the 5-day dosing period. Patients were followed up 21 days after completing the 5-day dosing period.

Dosing started at a dose of 0.15 μg $m^{-2}$ $day^{-1}$. This starting dose was selected as it was comparable with therapeutic doses of $1,25(OH)_2D_3$ and had been tolerated well by animals. Subsequent dose levels increased by 30–50% each time for the following patients and the maximum dose administered was 17 μg $m^{-2}$ $day^{-1}$.

### Compassionate treatment phase

Compassionate treatment for up to 1 year was allowed and commenced at the end of the per protocol treatment. The same dose level was to be given as that used in the per protocol phase, but this could be reduced if hypercalcaemia had been recorded during the per protocol phase or developed during compassionate treatment. In patients who became hypercalcaemic, EB 1089 treatment was stopped and the serum calcium allowed to return to normal before treatment recommenced.

### Calcium diet

All patients were seen by the hospital dietitian and commenced a low-calcium diet (an estimated 500 mg $day^{-1}$) at the start of the

© Cancer Research Campaign 1998

**Figure 2** Correlation of EB1089 dose in μg $m^{-2}$ with (**A**) serum-corrected calcium ($n = 32$), $P = 0.0267$; (**B**) urine calcium ($n = 28$), $P = 0.0001$; (**C**) parathormone ($n = 31$), $P = 0.0001$; (**D**) serum creatinine ($n = 34$) $P = 0.024$ (slope 0.0015). $P$ = values given are from linear regression on log ratio (day 6/day 1) by dose level (μg $m^{-2}$)

study. This was maintained during the per protocol and compassionate treatment phases. Patients were given a diet sheet when sent home and routinely questioned about compliance at follow-up. All other calcium and vitamin D supplements were stopped during the study.

### Assessment of response, toxicity and monitoring

At the start of the trial, patients were staged by means of clinical examination, chest radiograph, liver ultrasound and bone scan or skeletal survey. Computerized tomography (CT) scans were obtained when relevant to assess disease. Assessment of response

© Cancer Research Campaign 1998

was carried out according to standard UICC criteria (Hayward et al, 1987). Serum total calcium and blood pressure were monitored closely on the first and last days of the repeated dosing period. Blood was taken at 0, 1, 2, 3, 4, 6, 8 and 12 h after the first dose on these days, as well as daily during the period of administration. Clinical examination and electrocardiography were performed on day 2, on the first and last days of the repeated dosing period and 3 weeks later. Blood tests included routine haematology, biochemistry (including liver function tests) serum total calcium, albumin and PTH (Incstar intact PTH kit). Twenty-four-hour urine collections were made and calcium, phosphate, hydroxyproline (Gordeladze et al, 1978) and creatinine excretion were measured before receiving EB 1089 solution and were repeated on the day after the repeated dosing period and 3 weeks later.

In the compassionate treatment phase, staging with relevant scans was repeated every 8 weeks. During the compassionate phase, clinical examination and routine blood chemistry were performed either every 4 weeks or every 2 weeks if hypercalcaemia developed.

### Definition of hypercalcaemia and hypercalciuria

Corrected serum calcium was calculated using the formula [serum total calcium + [(40 – serum albumin)] × 0.02] mM $l^{-1}$. Hypercalcaemia was defined as a corrected serum calcium > 2.65 mM and severe hypercalcaemia as a corrected serum calcium > 2.80 mMl (or two consecutive values of > 2.75 mM). Hypercalciuria was defined as a 24 h urine calcium excretion > 7.5 mM).

During the per protocol phase, treatment with EB 1089 was withdrawn if a toxic event occurred. The parameters determining a toxic event were either severe hypercalcaemia, hypercalciuria (for the first 11 patients only) or a serious or unexpected adverse event.

During compassionate treatment with EB 1089, dosage reductions (about 50%) were made if severe hypercalcaemia developed, or clinical experience gained during the study suggested severe hypercalcaemia could be expected at any dose level. Patients were also to be withdrawn from treatment if they underwent disease progression, defined as a greater than 25% increase in one dimension of measurable lesion, the appearance of new lesions or significant clinical deterioration.

### Determination of maximum tolerated dose (MTD)

The MTD was determined by the continual reassessment method (CRM).

Patients were entered sequentially and allocated to a dose level, determined by the observed toxicity in the previously treated patients, using an extension of the CRM (O'Quigley et al, 1990; Moller, 1995). The method is based on a determination of an acceptable level of toxic response ($\pi$ = 40%) and the assumption that the dose–response curve of the probability of experiencing a severe toxicity could be described by a family of monotone functions: $f(x,\alpha)$, depending on the dose, $x$, and a parameter, $\alpha$, monotonously in both $x$ and $\alpha$. For each new observation of toxicity or no toxicity in a patient treated at dose $x$, the curve will be re-estimated based on all the available observations by estimating the parameter, $\alpha$, and the dose corresponding to the acceptable level of toxic response (MTD) will be calculated by: $p(MTD) = f(MTD, \alpha) = \pi$, and given to the next patient.

The family of dose–response curves for the dose determination is $f(z(x),\alpha) = ((\tanh(z(x))) + 1)/2)^{\alpha}$, where $z(x)$ is a linear function of $x$, normalizing the interval so that $f(z,1)$ will take values in the interval (0.05, 0.70) for the range of dose levels considered. The prior distribution of $\alpha$ before any patient entered is: $g(\alpha) = EXP(-\alpha)$. The distribution, $g(\alpha)$, will then be updated for each new observation.

### Statistical methods

The mean relative change from the baseline to the end of treatment after five repeated doses was analysed for all laboratory parameters including the calcium profile (total serum calcium, albumin-corrected serum calcium, urine calcium, serum creatinine and PTH) by using a $t$-test on the log ratio (day 6/day 1). The relationship between relative change and the dose was investigated using linear regression. The distribution of the patients' disease status and the rate of adverse events or hypercalcaemia at the end of treatment was correlated with the diagnosis and site(s) of metastasis and compared with dose levels using the $\chi^2$ test or Fisher's exact test.

The values at the end of compassionate treatment could not be used to evaluate drug safety as most of the patients left the compassionate treatment phase because of hypercalcaemia and/or medical deterioration. The analysis of dose dependency was performed using linear regression analysis and using analysis of variance on the time until leaving the study (after logarithmic transformation) in order to obtain normal distributions.

### Ethics

The study was conducted under clinical trials exemption and was approved by the Riverside Research ethics committee.

### RESULTS

Thirty-six patients all with progressive disease entered the study between May 1993 and June 1995. Patient demographics are shown in Table 1.

### Per protocol treatment

Eleven patients received the single day's dosing followed by the 5-day repeated dosing period at dose levels of 0.15–0.6 μg $m^{-2}$. Twenty-five patients received only the 5-day repeated dosing at dose levels of 0.9–17 μg $m^{-2}$. The different positive disease sites, as well as tumour sizes, were similarly distributed amongst the dose steps.

Neither hypercalciuria nor hypercalcaemia was recorded with one day's dosing. Eleven patients became hypercalcaemic during the 5-day repeated dosing. Four of these patients had severe hypercalcaemia at doses of 0.45, 12.5 and 17 (two) μg $m^{-2}$. The patient given 0.45 μg $m^{-2}$ became severely hypercalcaemic after 3 days, coincidental with a dramatic deterioration in her condition, including a marked fall in serum albumin, leading to an increase in corrected serum calcium, and died shortly after from the underlying disease.

Figure 2 shows the ratios of end of the 5-day repeated dosing period (day 6) to baseline (day 1) for the following parameters: corrected serum calcium, 24 h urine calcium, serum PTH, serum creatinine against EB 1089 dose in μg $m^{-2}$. $P$-values given are from linear regression on log ratio (day 6/day 1) by dose level (μg $m^{-2}$). There is a significant effect of EB 1089 on urinary calcium ($P = 0.0001$), and on the corrected serum calcium ($P = 0.027$), both increasing with dose. There is also a highly significant inverse

© Cancer Research Campaign 1998

**Table 2** Patients in compassionate treatment phase

| Sex | Age (years) | Primary tumour | WHO performance | Dose (µg m⁻²) | Days in compassionate treatment (CT) | Hypercalcaemia | Metastases | Days in CT until progression |
|---|---|---|---|---|---|---|---|---|
| F | 62 | Breast | 0 | 0.15 | 31 | | B O | 31 |
| F | 70 | Breast | 1 | 0.15 | 31 | | B O | 31 |
| F | 57 | Breast | 0 | 0.3 | 77 | | B O | 77 |
| F | 67 | Breast | 0 | 0.45 | 122 | | L B O | 122 |
| F | 71 | Breast | 0 | 0.6 | 168 | | O | 168 |
| F | 80 | Breast | 0 | 0.6 | 56 | | O | 56 |
| M | 75 | Colon | 0 | 1.2 | 91 | | O | [a] |
| F | 51 | Breast | 0 | 1.2 | 234 | Yes | B | 234 |
| F | 33 | Breast | 0 | 1.5 | 32 | | L O | 0 |
| F | 69 | Breast | 0 | 1.5 | 63 | Yes | L O | 42 |
| M | 44 | Appendix | 0 | 2.1 | 151 | | L O | 151 |
| M | 62 | Colon | 2 | 2.1 | 147 | Yes | O | 119 |
| M | 53 | Colon | 0 | 4 | 78 | Yes | O | 46 |
| F | 58 | Colon | 1 | 4 | 59 | | O | 0 |
| F | 34 | Breast | 0 | 5.5 | 105 | | O | 77 |
| M | 53 | Colon | 0 | 7 | 35 | Yes | L | 35 |
| F | 46 | Breast | 0 | 7 | 10 | Yes | L | 10 |
| F | 59 | Colon | 0 | 12.5 | 13 | Yes | O | [c] |
| F | 65 | Breast | 0 | 17, 7[b] | 66 | Yes | O | 31 |
| F | 45 | Breast | 0 | 17, 7, 4[c] | 196 | Yes | L B | 140 |
| F | 65 | Breast | 0 | 17, 7[b] | 28 | Yes | B | [d] |

[a]Patient withdrew from study. [b]Dose reductions due to hypercalcaemia. [c]Patient withdrawn with rising calcium. [d]Patient withdrawn due to hypercalcaemia. B, bone; L, liver; O, other

**Figure 3** Hypercalcaemic patients during the compassionate treatment phase. Values after the dotted lines indicate end-of-treatment measurements when treatment continued longer than the 120 days shown. *Two patients with similar readings at these points

 © Cancer Research Campaign 1998

relationship with serum parathyroid hormone level ($P = 0.0001$) and the relationship between EB 1089 and serum creatinine also achieved significance ($P = 0.024$).

### Compassionate treatment

Twenty-one patients received compassionate treatment for between 10 and 234 days (mean 90 ± 62 days) (Table 2). Eleven patients remained normocalcaemic throughout compassionate treatment. Ten patients became hypercalcaemic, which was severe in six (Figure 3). It was transient in one case and resolved without any dose changes. Of the remaining nine patients, four became hypercalcaemic at doses they had tolerated during the 5-day dosing period, and five patients had also developed hypercalcaemia during the 5-day dosing period. In these nine patients, hypercalcaemia was not significantly related to the diagnosis [six (24%) breast cancer, three (27%) colorectal cancer] or the presence of bone metastasis [five (33%) with bone metastases, four (19%) without]. Only one of these (receiving 12.5 μg $m^{-2}$) was symptomatic: she had bilateral hydronephroses with no known cause, but had normal renal function. Hypercalcaemia usually resolved within 7 days of ceasing treatment with EB 1089.

### Estimate of MTD

The dose–response curve with respect to toxicity was estimated after the study was completed, based on the last 11 patients who received the 5-day repeated dosing at dose levels 7–17 μg $m^{-2}$. The transformation of log dose was: $Z(x) = (\log_{10}(x) - 0.84)\ 1.89/0.63 - 1.47$ and the estimated MTD was 17.5 μg $m^{-2}$ (Figure 4). An additional dose–response curve was estimated, based on all 25 patients treated at dose levels ≥ 0.9 μg $m^{-2}$ and for all treatment periods, including compassionate treatment. The transformation of log dose was changed to cover the dose interval: [0.9–3.0 μg $m^{-2}$]: $Z(x) = (\log_{10}(x) - 0.05)\ 1.89/1.53 - 1.47$. On this basis the estimated MTD was 7 μg $m^{-2}$ (Figure 4).

### Anti-tumour effects

No clear-cut anti-tumour effects were seen in this study. Eighteen patients received compassionate treatment for at least 30 days: 12 at the lower doses of 0.15–3.0 μg $m^{-2}$ and six at doses between 4.0 and 17 μg $m^{-2}$. Six patients showed stabilization of disease in excess of 3 months. These patients received the following doses: 0.45, 0.6, 1.2, 2.1, 2.1 and 17 subsequently reduced to 7 μg $m^{-2}$ then 4 μg $m^{-2}$. Four had breast cancer and two had colorectal cancer.

### Safety monitoring

EB 1089 treatment had no effect on systolic or diastolic blood pressure, heart rate or ECG. There was no effect of EB 1089 on the indices of haematopoietic or hepatic function monitored. During per protocol treatment, EB 1089 did not affect urinary excretion of creatinine, phosphate and hydroxyproline. During compassionate treatment there was no effect on laboratory parameters, apart from on serum calcium.

### Adverse events

Three patients had non-calcaemic adverse events during the 5-day dosing and follow-up. These were pain and tiredness (one), dizziness

**Figure 4** Estimated dose response curves for the probability of toxicity. ○Estimate of MTD based on 5-day dosing. ★Estimate of MTD based on all treatment periods

(one) and left-bundle branch block (one) in a patient who was found subsequently to have ischaemic heart disease. During compassionate treatment, eight non-calcaemic adverse events were recorded in six patients, two of whom had adverse events during the 5-day dosing period and comprised nausea and vomiting (two), pain (two), increased pleural effusion (one), rising alkaline phosphatase (one) and raised gamma-glutamyltranspeptidase (one). None of these events was clearly related to EB 1089 treatment.

### DISCUSSION

To our knowledge, this is the first reported study of a systemically administered synthetic analogue of vitamin D in the therapy of human cancer. Administration of EB 1089 solution was not associated with any toxicity clearly attributed to the drug other than those abnormalities associated with calcium metabolism.

When EB 1089 solution was administered for only 5 days, treatment-related hypercalcaemia developed in ten patients. Using the data from the higher dose levels only, the MTD was determined as 17.5 μg $m^{-2}$. However, prolonged treatment with EB 1089 solution at such doses was invariably associated with hypercalcaemia. Of the ten patients who became hypercalcaemic on compassionate treatment, four did so at doses they had tolerated for 5 days. Delayed hypercalcaemia was a feature of prolonged EB 1089 (longer than 5 days). Hypercalcaemia resolved, usually within 7 days, when treatment was withdrawn. The best estimate of the MTD for prolonged treatment with EB 1089 solution from this study is around 7 μg $m^{-2}$. Preliminary data from phase II trials currently underway confirm a dose range of 10–20 μg daily. This dose is significantly higher than that recorded for $1,25(OH)_2D_3$, when hypercalcaemia is invariably observed at doses of 2 μg (Vieth, 1990).

It is encouraging that the expected decrease in calcaemic activity of EB 1089 relative to that of its parent compound, ($1,25(OH)_2D_3$), has translated into the clinical setting. Certainly the dose estimated to be tolerable to patients (7 μg $m^{-2}$, corresponding to around 0.2 μg $kg^{-1}$ in man) is similar to, or greater than, those shown to have anti-tumour tumour effects in animal models (Haq et al, 1993; Colston, 1994; DL Morris, personal communication).

Pharmacokinetic studies were not conducted in this study. No suitable assay was available to measure EB 1089 in urine or serum. However, the significant correlation of EB 1089 dose with serum calcium and inverse correlation with serum PTH indicates adequate bioavailability of the drug.

The relationship between EB 1089 dose and serum creatinine seen with acute (5 day) doses is not explained. However, this funding is in accordance with other trials involving calcitriol, alphacalcidol and other analogues. These studies report reversible increases in serum creatinine, with other changes in measurements of renal function, such as creatinine clearance, not being demonstrated (Tvedegaard et al, 1988; Bertoli et al, 1990). Creatinine clearance was not determined in our study. However, it is reassuring to note that when EB 1089 was given for prolonged periods during compassionate treatment there was no change in serum creatinine.

We failed to observe any anti-tumour effects in our patients. However, all patients had received anti-cancer therapy previously and many of the breast cancer patients had been given more than three different therapeutic regimens in the past. It is reasonable to expect that the 'differentiation agents', such as EB 1089, are unlikely to have a measurable effect in this setting. Responses to these agents may be more likely in the earlier stages of disease or in patients with minimal disease.

Recent research on new synthetic vitamin D analogues clearly shows the possibility of developing derivatives that separate potent modulatory effects on cell growth and differentiation and effects on calcium homeostasis, and has established an exciting potential for these compounds as therapeutic agents in malignancy. This study described the administration of one of these agents, EB 1089, to cancer patients. Adverse events were limited to dose-dependent predictable effects on calcium metabolism, and the drug can be given at doses similar to the doses that we judge, on the basis of animal studies, are needed for anti-tumour activity.

### ACKNOWLEDGEMENTS

TG is supported by the MRC and RCC by a CRC Programme Grant. We would like to thank Dr Richard Epstein for constructive criticism of the manuscript and Members of the CRC Phase I/II Committee for supporting the initial toxicology and Mrs Jean Sterling for typing the manuscript.

### REFERENCES

Abe E, Miyaura C, Sakagami H, Takeda M, Konno K, Yamazaki T, Yoshiki S and Suda T (1981) Differentiation of mouse myeloid leukemia cells induced by 1 alpha, 25-dihydroxyvitamin D3. *Proc Natl Acad Sci USA* **78**: 4990–4994

Abe J, Morikawa M, Miyamoto K, Kaiho S, Fukushima M, Miyaura C, Abe E, Suda T and Nishii Y (1987) Synthetic analogues of vitamin D3 with an oxygen atom in the side chain skeleton. A trial of the development of vitamin D compounds which exhibit potent differentiation-inducing activity without inducing hypercalcaemia. *Febs Lett* **226**: 58–62

Akhter J, Chen X, Bowrey P, Bolton EJ and Morris DL (1997) Vitamin $D_3$ analog, EB 1089, inhibits growth of subcutaneous xenografts of the human colon cancer cell line, LoVo, in a nude mouse model. *Dis Colon Rectum* **40**: 317–321

Bagot M, Grossman R, Pamphile R, Binderup L, Charue D, Revuz J and Dubertret L (1994) Additive effects of calcipotriol and cyclosporine A: from in vitro experiments to in vivo applications in the treatment of severe psoriasis. *CR Acad Sci Iii* **317**: 282–286

Bar SZ, Teitelbaum SL, Reitsma P, Hall A, Pegg LE, Trial J and Kahn AJ (1983) Induction of monocytic differentiation and bone resorption by 1,25-dihydroxyvitamin D3. *Proc Natl Acad Sci USA* **80**: 5907–5911

Bell NH (1985) Vitamin D-endocrine system. *J Clin Invest* **76**: 1–6

Berger U, Wilson P, McClelland RA, Colston K, Haussler MR, Pike JW and Coombes RC (1987) Immunocytochemical detection of 1,25-dihydroxyvitamin D3 receptor in breast cancer. *Cancer Res* **47**: 6793–6795

Bertoli M, Luisetto G, Ruffatti A, Urso M and Romagnoli G (1990) Renal function during calcitriol therapy in chronic renal failure. *Clin Nephrol* **33**: 98–102

Binderup L and Bramm E (1988) Effects of a novel vitamin D analogue MC 903 on cell proliferation and differentiation in vitro and on calcium metabolism in vivo. *Biochem Pharmacol* **37**: 889–895

Bower M, Colston KW, Stein RC, Hedley A, Gazet JC, Ford HT and Coombes RC (1991) Topical calcipotriol treatment in advanced breast cancer. *Lancet* **337**: 701–702

Brehier A and Thomasset M (1988) Human colon cell line HT-29: characterisation of 1,25-dihydroxyvitamin D3 receptor and induction of differentiation by the hormone. *J Steroid Biochem* **29**: 265–270

Carlberg C (1995) Mechanisms of nuclear signalling by vitamin $D_3$. Interplay with retinoid and thyroid hormone signalling. *Eur J Biochem* **231**: 517–527

Colston K, Colston MJ and Feldman D (1981) 1,25-dihydroxyvitamin D3 and malignant melanoma: the presence of receptors and inhibition of cell growth in culture. *Endocrinology* **108**: 1083–1086

Colston KW, Mackay AG, James SY, Binderup L, Chander S and Coombes RC (1992) EB1089: a new vitamin D analogue that inhibits the growth of breast cancer in vivo and in vitro. *Biochem Pharmacol* **44**: 2273–2280

Eisman JA, Barkla DH and Tutton PJ (1987) Suppression of in vivo growth of human cancer solid tumor xenografts by 1,25-dihydroxyvitamin D3. *Cancer Res* **47**: 21–25

Elstner E, Lee YY, Hashiya M, Pakkala S, Binderup L, Norman AW, Okamura WH and Koeffler HP (1994) 1 alpha,25-dihydroxy-20-epi-vitamin D3: an extraordinarily potent inhibitor of leukemic cell growth in vitro. *Blood* **84**: 1960–1967

Elstner E, Linker-Israeli M, Umiel T, Le J, Grillier I, Said J, Shintaku IP, Krajewski S, Reed JC, Binderup L and Koeffler HP (1996) Combination of a potent 20-epi-vitamin D3 analogue (KH10060) with 9-*cis*-retinoic acid irreversibly inhibits clonal growth, decreases bcl-2 expression, and induces apoptosis in HL-60 leukemic cells. *Cancer Res* **56**: 3570–3576

Frampton RJ, Omond SA and Eisman JA (1983) Inhibition of human cancer cell growth by 1,25-dihydroxyvitamin D3 metabolites. *Cancer Res* **43**: 4443–4447

Gordeladze JO, Halse J, Djooseland O and Haugen HNA (1978) Simple procedure for the determination of hydroxyproline in urine and bone. *Biochem Med* **20**: 23–30

Gram J, Junker P, Nielsen HK and Bollerslev J (1996) Dose response effect of short-term calcitriol treatment on bone and mineral metabolism in normal males. *Bone* **18**: 539–544

Hannah SS and Norman AW (1994) 1 $\alpha$,25(OH)$_2$ vitamin $D_3$-regulated expression of the eukaryotic genome. *Nutr Rev* **52**: 376–382

Hansen CM, Frandsen TL, Brunner N and Binderup L (1994) 1 alpha,25-dihydroxyvitamin D3 inhibits the invasive potential of human breast cancer cells in vitro. *Clin Exp Metastasis* **12**: 195–202

Hayward JL and Rubens RD (1987) UICC Multidisciplinary Project on breast cancer. Management of early and advanced breast cancer. *Int J Cancer* **39**: 1–5

Haq M, Kremer R, Goltzman D and Rabbani SA (1993) A vitamin D analogue (EB 1089) inhibits parathyroid hormone-related peptide production and prevents the development of malignancy-associated hypercalcaemia in vivo. *J Clin Invest* **91**: 2416–2422

Hosomi J, Hosoi J, Abe E, Suda T and Kuroki T (1983) Regulation of terminal differentiation of cultured mouse epidermal cells by 1 alpha,25-dihyroxyvitamin D3. *Endocrinology* **113**: 1950–1957

James SY, Mackay AG, Binderup L and Colston KW (1994) Effects of a new synthetic vitamin D analogue, EB 1089, on the oestrogen-responsive growth of human breast cancer cells. *J Endocrinol* **141**: 555–563

James SY, Mackay AG and Colston KW (1995) Vitamin D derivatives in combination with 9-*cis*-retinoic acid promote active cell death in breast cancer cells. *J Mol Endocrinol* **14**: 391–394

Kragballe K (1992) Vitamin D analogues in the treatment of psoriasis. *J Cell Biochem* **49**: 46–52

Kragballe K, Gjertsen BT, De Hoop D, Karlsmark T, van de Kerkhof PC, Larko O, Nieboer C, Roed Petersen J, Strand A and Tikjob G (1991) Double-blind, right/left comparison of calcipotriol and betamethasone valerate in treatment of psoriasis vulgaris. *Lancet* **337**: 193–196

Majewski S, Szmurlo A, Marczak M, Jablonska S and Bollag W (1993) Inhibition of tumor cell-induced angiogenesis by retinoids, 1,25-dihydroxyvitamin D3 and their combination. *Cancer Lett* **75**: 35–39

Mangelsdorf DJ, Thummel C, Beato M, Herrlich P, Schutz G, Umesono K, Blumberg B, Kastner P, Mark M, Chambon P and Evans RM (1995) The nuclear receptor superfamily: the second decade. *Cell* **83**: 835–839

© Cancer Research Campaign 1998

Moller S (1995) An extension of the continual reassessment methods using a preliminary up-and-down design in dose finding study in cancer patients, in order to investigate a greater range of doses. *Stat Med* **14**: 911–922

Norman AW, Zhou JY, Henry HL, Uskokovic MR and Koeffler HP (1990) Structure-function studies on analogues of 1 alpha,25-dihydroxyvitamin D3: differential effects on leukemic cell growth, differentiation, and intestinal calcium absorption. *Cancer Res* **50**: 6857–6864

O'Quigley J, Pepe M and Fisher L (1990) Continual reassessment method: a practical design for phase 1 clinical trials in cancer. *Biometrics* **46**: 33–48

Ostrem VK, Lau WF, Lee SH, Perlman K, Prahl J, Schnoes HK and DeLuca HF (1987) Induction of monocytic differentiation of HL-60 cells by 1,25-dihydroxyvitamin D analogs. *J Biol Chem* **262**: 1464–1471

Skowronski RJ, Peehl DM and Feldman D (1993) Vitamin D and prostate cancer: 1,25-dihydroxyvitamin D3 receptors and actions in human prostate cancer cell lines. *Endocrinology* **132**: 1952–1960

Thomas MG, Tebbutt S and Williamson RC (1992) Vitamin D and its metabolites inhibit cell proliferation in human rectal mucosa and a colon cancer cell line. *Gut* **33**: 1660–1663

Tvedegaard E and Madsen S (1988) Alphacalcidol and renal function in normal subjects. *Min Electrolyte Metab* **14**: 158–162

Vieth R (1990) The mechanisms of vitamin D toxicity. *Bone Min* **11**: 267–272

Zhou JY, Norman AW, Chen DL, Sun GW, Uskokovic M and Koeffler HP (1990) 1,25-dihydroxy-16-ene-23-yne-vitamin D-3 prolongs survival time of leukemic mice. *Proc Natl Acad Sci USA* **87**: 3929–3932

© Cancer Research Campaign 1998